# Design, Commissioning, and Performance Assessment of a Lab-Scale Bubble Column Reactor for Photosynthetic Biogas Upgrading with *Spirulina platensis*

**DOI:** 10.1021/acs.iecr.0c05974

**Published:** 2021-04-08

**Authors:** Archishman Bose, Richard O’Shea, Richen Lin, Jerry D. Murphy

**Affiliations:** † Environmental Research Institute, MaREI Centre, University College Cork, Cork T23 XE10, Ireland; ‡ School of Engineering, University College Cork, Cork T23 XE10, Ireland

## Abstract

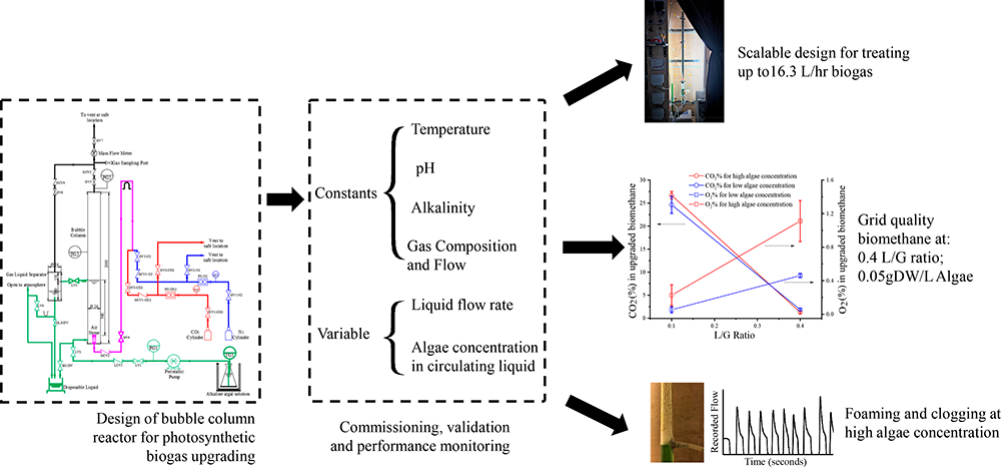

The two-step bubble column-photobioreactor photosynthetic biogas upgrading system can enable simultaneous production of biomethane and value-added products from microalgae. However, due to the influence of a large number of variables, including downstream processes and the presence of microalgae, no unanimity has been reached regarding the performance of bubble column reactors in photosynthetic biogas upgrading. To investigate this further, the present work documents in detail, the design and commissioning of a lab-scale bubble column reactor capable of treating up to 16.3 L/h of biogas while being scalable. The performance of the bubble column was assessed at a pH of 9.35 with different algal densities of *Spirulina platensis* at 20 °C in the presence of light (3–5 klux or 40.5–67.5 μmol m^–2^ s^–1^). A liquid/gas flow (L/G) ratio of 0.5 allowed consistent CO_2_ removal of over 98% irrespective of the algal density or its photosynthetic activity. For lower concentrations of algae, the volumetric O_2_ concentration in the upgraded biomethane varied between 0.05 and 0.52%, thus providing grid quality biomethane. However, for higher algal concentrations, increased oxygen content in the upgraded biomethane due to both enhanced O_2_ stripping and the photosynthetic activity of the microalgae as well as clogging and foaming posed severe operational challenges.

## Introduction

### Photosynthetic Biogas Upgrading

Conventional physicochemical biogas upgrading technologies such as water scrubbing and pressure swing absorption continue to consume a significant amount of energy (up to 6% of the energy content in biogas).^1^ In addition, it also results in a high cost of biomethane, necessitating the need for incentives to achieve financial sustainability.^2^ As an alternative, biological biogas upgrading technologies are being investigated to increase the sustainability of biomethane derived from biogas by reducing costs and energy demands.^1^ Photosynthetic biogas upgrading is a novel biogas upgrading technology that can be employed to remove CO_2_ and H_2_S in biogas by absorption in a carbonate–bicarbonate-based alkaline algal medium.^3,4^ The absorbed CO_2_ would allow the cultivation of microalgae for food, feed, and/or energy.^5^ As a consequence, the resulting biomethane could be both economically and environmentally more beneficial than that obtained through traditional biogas upgrading systems.^5,6^ The two-step bubble column-photobioreactor (PBR) photosynthetic biogas upgrading configuration is currently under assessment by the scientific community for optimization of both biogas upgrading in a bubble column and growth of microalgae in a PBR.^7,8^

### Significance of Operation and Design of the Bubble Column

Effective bubble column operation in photosynthetic biogas upgrading must achieve continuous grid quality biomethane; this typically requires biomethane with CO_2_ and O_2_ concentrations below 2.5 and 1% on a volume basis, respectively.^9,10^ PBRs should then ensure rapid and effective CO_2_ utilization by microalgae to achieve a sustainable biogas upgrading facility utilizing microalgae. Optimization studies for CO_2_ removal in bubble column reactors with carbonate–bicarbonate solutions in the absence of microalgae are available in the literature as a stand-alone setup.^11,12^ However, such optimal operational conditions in the bubble column may not be ideal for microalgae cultivation. For example, the requirement of a high temperature (above 50 °C)^13^ and a high pH (12 and above)^14^ is fatal to most microalgae species.^8^ At a temperature and a pH below 35 °C and 10, respectively, CO_2_ absorption rates drop significantly.^14^ Therefore, the bubble columns for photosynthetic biogas upgrading are usually of a large aspect ratio (ratio of height to diameter, also termed length to diameter or *L*/*D* ratio) and employ low gas flow rates. This in turn results in a high empty bed residence time or EBRT [min] (signifying the time taken by the gas to traverse the length of the bubble column in the absence of any liquid medium), typically ranging between 15 and 90 min (Table 1). Typical EBRTs for industrial applications are in the range of 3–6 min,^15^ and as such, this would potentially somewhat limit the scale-up and industrial application of not only the bubble column but also the photosynthetic biogas upgrading technology. In addition, although present in minor quantities (<1%), H_2_S in biogas could affect the performance of CO_2_ absorption in inadequately designed bubble columns for photosynthetic biogas upgrading. To highlight this, Meier et al. (2018)^3^ observed no significant change in CO_2_ removal efficiency (98%) in the presence (1800–3500 ppm_v_) and absence of H_2_S in the biogas when the bubble column pH was maintained around 8.7. However, Bahr et al. (2014),^15^ controlling the bubble column inlet pH at 8.5, noticed a drop in the CO_2_ removal efficiency from 95 ± 3 to 89 ± 5% when the H_2_S content in the biogas increased from 0 to 500 ppm_v_. However, when it was further increased to 1500 ppm_v_, no further drop in the CO_2_ removal efficiency was noticed. In both the studies, an almost complete H_2_S removal was recorded. Therefore, ensuring conditions in the bubble column for sufficient CO_2_ removal is required to allow simultaneous H_2_S removal with a minor influence on the absorption of CO_2_ and bubble column performance. The absorbed H_2_S in the form of sulfates, besides providing a valuable nutrient supplement, was found to have no influence on the growth rates of microalgae and design of PBRs.^3^

**Table 1:** Scale and Design Factors of Bubble Columns Applied for Photosynthetic Biogas Upgrading in the Recent Literature^a^

		column dimensions					operation parameters								optimal biomethane composition (%)	
references	scale	diameter (cm)	aspect ratio^c^	volume (L)	mode	gas flow rate (LPH)	L/G ratio	superficial gas velocity (cm/s)	EBRT (min)	pH	alkalinity (mg-IC/L)	temperature (°C)	*X*_algae_ (g/L)	light	CO_2_ (%)	O_2_ (%)
Rodero et al. (2020)^23^	L	NR	NR	2.5	C	3	0.5	NR	NR	10	3152–3814	28 ± 2	varies	CLD	0.6–1	0.1 ± 0.1
Rodero et al. (2020)^21^	SI	NR	NR	150	CC	143–420	0.8–2.3	NR	NR	9.05–9.50	1907 ± 109	24.2 ± 2	NR^b^	OP	2.4	≤1
Rodero et al. (2019)^24^	L	NR	NR	2.5	C	3.6–9	≤1.1	NR	NR	8.5–10	100–1500	15 and 35	NR^b^	NR	1.5 (3.6 LPH gas flow)	≤1
Rodero et al. (2019)^25^	SI	SI	SI	150	CC	274–459	1.2–3.5	NR	NR	7.3–8.9	NR	12.4–23.5	NR^b^	OP	0.3 (L/G 3.5)	7.4 ± 0.4 (L/G 3.5)
Marin et al. (2018)^17^	P	4.4	37.5	2.5	C	3.12	1	0.057	48.25	9.4	1663–4138	9.1–24.4	NR^b^	OP	0.7–11.9	0–3.4
Rodero et al. (2018)^20^	L	4.4	37.5	2.5	C	4.9	0.5	0.089	30.89	7.2–11.5	100–1500	15 and 35	NR^b^	CLD	At pH 10.5: 0.9 ± 0.3 (1467 ± 115 g-IC/L), 18.4 ± 1 (505 ± 57 g-IC/L)	0–0.2
Posadas et al. (2017)^26^	P	4.4	37.5	2.5	C	3.12	0.5–5	0.057	48.25	8.8–9.8	267–2660	20–23.8	NR^b^	OP	8.8	0.7–1.1
Toledo-Cervantes et al. (2017)^16^	L	4.4	37.5	2.5	C/CC	2.4	0.3–1	0.044	62.5	10.2 ± 0.5	1500 ± 168	23.8 ± 1.7	NR^b^	CLD	0.4 ± 0.3 (C)	0.7 ± 0.4 (C)
Meier et al. (2017)^9^	L	1.2	2500	NR	CC	2.08	0.6	0.512	97.66	7.3	660–880	20–28	varies	CLD	2–4.5	≤1
Franco-Morgado et al. (2017)^27^	L	1.9	NR	0.35	CC	0.65	5	NR	NR	9.3–9.7	1130 ± 0.09	16–28	NR^b^	CLD	0–0.3	2.6
Posadas et al. (2015)^28^	L	4.4	37.5	2.5	C	1.85 ± 0.07	10.7 ± 0.4	0.034 ± 0.001	∼80	7.9	66–126	23 ± 1	NR^b^	CLD	6.6 ± 0.7	≤1.2
Serejo et al. (2015)^29^	L	4.4	37.5	2.5	C	0.5–67	varies	0.006–0.033	83.33–458.33	7.9	63–82	20.9–26.4	NR^b^	CLD	0.9–2.1 (L/G ≥ 15)	3 ± 1 (L/G ≥ 15)
Meier et al. (2015)^4^	L	2	110	0.7	C	0.09	2.33	0.055	66.67	7.7 ± 0.2	88–176	20 ± 2	varies	CLD	1.9	1.2

a The acronyms are as follows: L: laboratory scale; P: pilot scale; SI: semi-industrial scale; NR: not reported; C: co-current; CC: counter-current; LPH: litres per hour; IC: inorganic carbon; *X*_algae_: algae concentration; OP: open pond; CLD: controlled light and dark cycles.

b Algal concentration although not explicitly reported, the algae were circulated in the bubble column after harvesting.

c Aspect ratio signifies the height-to-diameter ratio of the bubble column.

Stripping of dissolved oxygen from the algal liquid during biogas upgrading is also a major concern. Often, this leads to oxygen concentrations in the upgraded biomethane beyond permissible limits for grid injection.^8,16^ Additional technological bottlenecks, such as the fluctuation in biomethane composition due to the diurnal^9^ and seasonal^17^ variations in the PBR, especially open ponds, must also be overcome to achieve practical applications of photosynthetic biogas upgrading. Thus, optimization and robust operation of the bubble column for biogas upgrading in conjunction with photosynthetic biogas upgrading require further analysis and research.

Optimization of bubble columns for industrial applications is often carried out in a lab-scale setup with a corresponding scale-up strategy.^18,19^ The primary challenge for such studies is the effective prediction of the scaled-up performance of the bubble column. This is due to the difference in either/or (i) heat- and mass-transfer characteristics; (ii) mixing and flow characteristics; (iii) chemical kinetics of the reacting system for the two separate scales (i.e., laboratory and industrial).^18,19^ Therefore, despite the simplistic design of the bubble column, the choice of different design parameters needs to be validated and appropriate scale-up criteria must be chosen to predict the industrial operation of the bubble column. In addition, the chosen design factors must also be robust and allow for flexibility in operation apart from providing essential information on techno-economic and environmental aspects^17^ of the operation of the bubble column.

### Current Trends in Bubble Column Design for Photosynthetic Biogas Upgrading

Photosynthetic biogas upgrading utilizing a separate bubble column depends on multiple factors such as biogas composition, pH, gas and liquid flow rates, concentration of the algae, temperature, and alkalinity as well as the microalgae species and cultivation conditions.^8,16,20^ Numerous studies have been undertaken to advance the aforementioned technology; however, the results and conclusions have varied significantly. This can be attributed to the large variability in design factors, operation parameters, and system configurations selected in the studies, summarized in Table 1.

For instance, while Toledo-Cervantes et al. (2017)^16^ concluded that a liquid to gas flow rate (L/G ratio) ratio of less than 1 is essential to achieve grid quality biomethane, Rodero et al. (2020)^21^ reported 14.1 % CO_2_ concentration in upgraded biomethane at an L/G ratio of 0.8. The aim to minimize both CO_2_ and O_2_ simultaneously has also led to contradicting values of process parameters. For example, while a pH greater than 9 is essential to ensure adequate CO_2_ removal, a higher pH also appears to increase O_2_ stripping into biomethane.^8^ In another instance, increasing algal concentration has been shown to improve bubble column hydrodynamics.^22^ This could enhance both CO_2_ removal and oxygen stripping. In fact, the presence of photosynthesizing microalgae could increase the oxygen content in the upgraded biomethane further. However, in a recent study, Rodero et al. (2020)^23^ obtained no statistically significant difference in CO_2_ removal rate with increasing algal concentration in the bubble column. The impact on oxygen stripping was also not reported. A focused assessment of bubble column operations could therefore be considered essential to optimize biogas upgrading, which in turn would assist in advancing the overall photosynthetic biogas upgrading technology.

### Objective

To study in detail the performance and hence the opportunity to optimize photosynthetic biogas upgrading, a lab-scale bubble column unit was designed and commissioned. In the present work, rather than optimizing the bubble column performance, its design and commissioning have been studied and documented. The performance of the bubble column has been evaluated under different operating conditions with the following objectives:

i. validating the design and performance of a lab-scale bubble column reactor for photosynthetic biogas upgrading,
ii. identifying and resolving challenges to operating and assessing the performance of such facilities on both the laboratory and industrial scales,
iii. studying the impacts of algal concentration and photosynthetic activity on reactor performance, and
iv. developing a scale-up perspective and integration with microalgae cultivation systems.

## Design and Evaluation of the Bubble Column for Photosynthetic Biogas Upgrading

### Working Range and the Operating Medium

A bubble column for photosynthetic biogas upgrading should be able to operate with a CO_2_ and H_2_S content in the inlet biogas between 20 and 50% and between 0 and 10,000 ppm, respectively. A homogeneous flow regime, characterized by uniform flow and smaller bubbles (∼3 mm diameter^30^), is preferable for CO_2_ absorption due to the availability of a larger surface area and improved hydrodynamic and hydraulic performance.^12,31^*Spirulina platensis* would be one of the most favorable microalgae species of choice for biogas upgrading, as discussed in a previous work by the authors.^8^ As such, the maximum working pH and temperature of the bubble column should be limited to 11 and 37 °C to avoid fatal consequences for *S. platensis*.^32^ Finally, the bubble column was operated near atmospheric pressures for ease of operation and facilitating integration with the open pond and closed PBRs alike.

### Working Principle for CO_2_ Absorption

In a carbonate–bicarbonate buffer solution, the carbonate ions dissociate in water to locally form hydroxyl ions via eq 1(33)
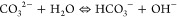
1

CO_2_ absorption in such a solution (typically above pH 8) can then be described in two steps, the irreversible hydration equation (eq 2) producing bicarbonates and the instantaneous reversible proton-transfer reaction (eq 3) yielding back carbonates.^33,34^ The hydration equation, driven by locally formed hydroxyl ions, is usually considered the rate-determining step for the CO_2_ absorption process^34,35^
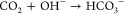
2
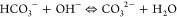
3

The overall absorption of CO_2_ in the carbonate–bicarbonate buffer solution can thus be described as a combination of eqs 1 and 2 as per eq 4(8,33,35)
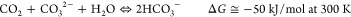
4

## Design and Configuration of the Lab-Scale Bubble Column

### Description of the Overall Setup

**Figure 1:**
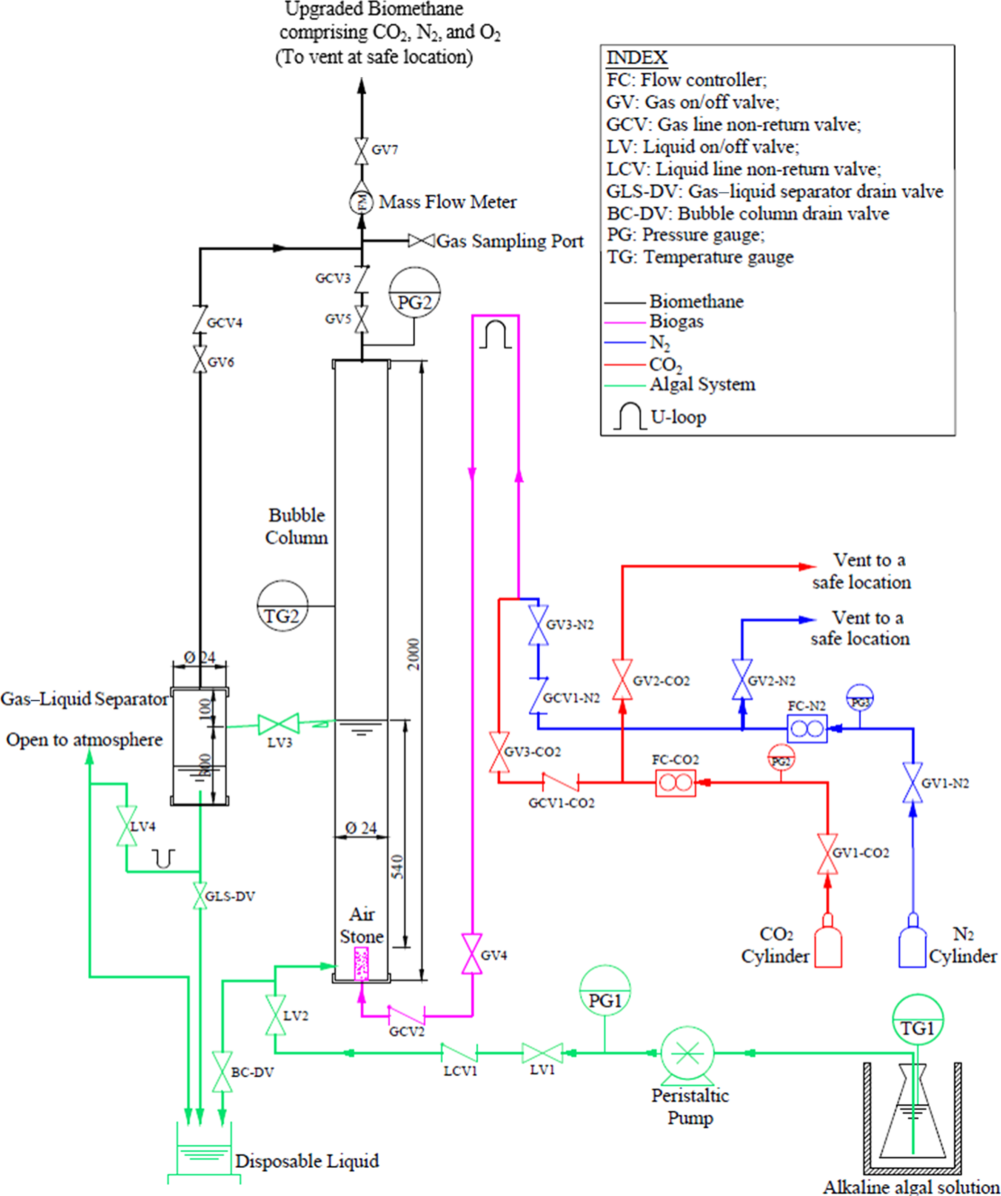
Process flow diagram and design details of the lab-scale bubble column for photosynthetic biogas upgrading (not to scale).

A lab-scale bubble column with a 24 mm inner diameter (*D*_BC_) and 2 m high was built using a clear acrylic tube. The co-current flow configuration was selected as per recommendations published by Toledo-Cervantes et al. (2017).^16^ A cylindrical diffuser (25 mm long; 18 mm diameter) was vertically mounted at the bottom of the column to sparge the gas into the bubble column. All liquid heights were measured from the top of the sparger. Similar to recently studied lab-scale bubble columns for photosynthetic biogas upgrading,^16,17^ the column height (*H*_BC_) selected for startup and commissioning was 540 mm (an aspect ratio or *L*/*D* ratio of 22.5). This was implemented by tapping a liquid outlet at the desired height, as indicated in Figure 1.

**Figure 2:**
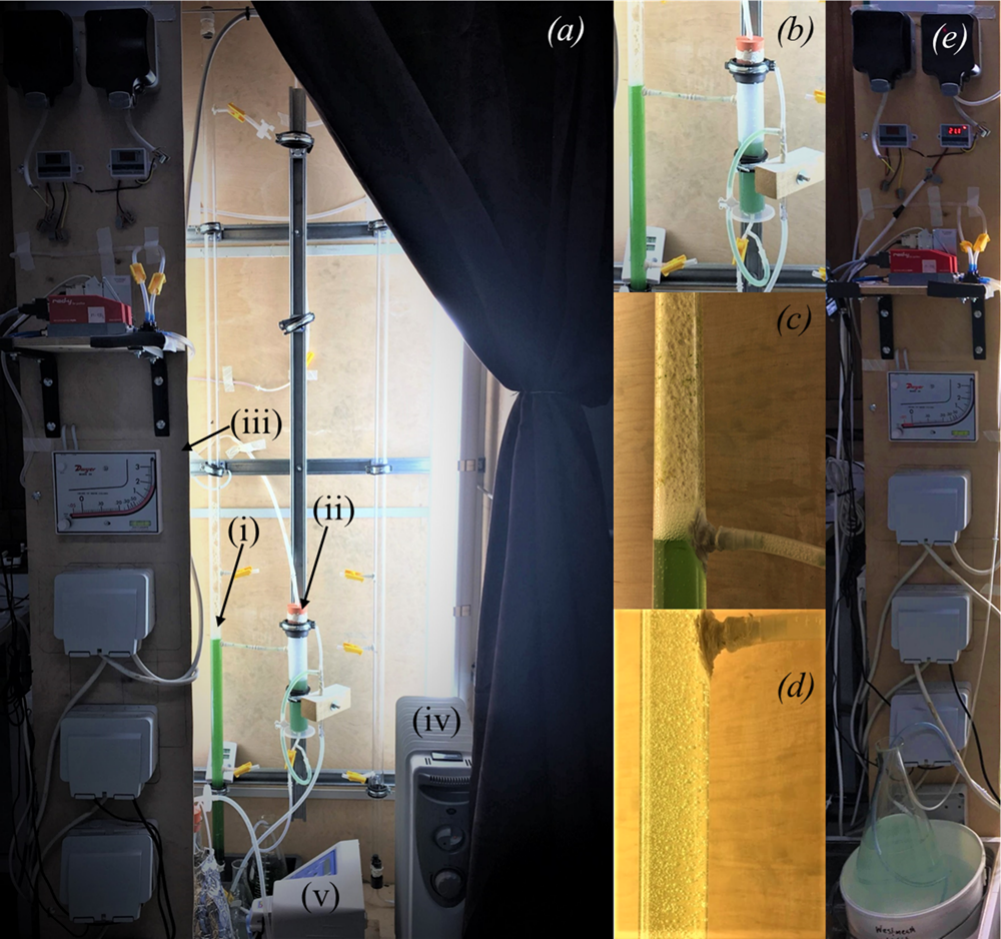
Pictorial representation of the experimental setup for photosynthetic biogas upgrading with (a) overall bubble column reactor setup within a temperature- and light-controlled housing; (b) details of the gas–liquid separator with a U-loop to prevent emptying by syphoning and also adjust the outlet liquid flow rate as per the inlet flow rate; (c) example of foaming observed during operation with a high algae concentration; (d) representation of the homogeneous flow regime characterized by a uniform bubble size under a low algal concentration; (e) details of the control panel with the condensate trap placed within an ice bath (bottom of the figure). Additional details marked in figure (a) represent (i) the bubble column (additional tapping points for future experiments); (ii) the gas–liquid separator; (iii) control panel with gas flow controllers, a mass flow meter, a manometer gauge, and the switches for temperature control; (iv) the heater; and (v) the peristaltic pump.

The entire setup was housed in a temperature-controlled cabinet, capable of operating between 20 and 35 °C. Heating and cooling were provided by an electric heater and an extractor fan, respectively, which were connected to thermostatic controllers with temperature probes placed near the bubble column. The cabinet was also fitted with two cool white fluorescent lights to provide an illumination between 2 and 6 klux (27–81 μmol m^–2^ s^–1^). A pictorial representation of the experimental setup is shown in Figure 2a.

### Inlet Configuration

Co-current operations mandated both the biogas and the liquid (alkaline algal solution) to be injected from the bottom of the bubble column. Liquid circulation into the bubble column at the desired flow rates was obtained using a VWR AU-UPC-EZ programmable peristaltic pump [2–70 mL/min with a 1.6 mm (ID) tube]. The tube on the suction side of the pump was fitted with a fine mesh cloth at its mouth to prevent algal clumps from flowing into the bubble column. The mesh cloth provided a fair replication of a foot valve, widely used in the industry at the mouth of the suction line of a liquid circulating pump to screen dirt, clumps and other particles. The gas supply was controlled by separate N_2_ and CO_2_ flow controllers (red-y smart controller GSC, Vögtlin Instruments GmbH). This follows the recommendations of Serejo et al. (2015)^29^ where N_2_ was used to replace methane to avoid explosion hazards in experimental setups. To prevent backflow of the liquid into the inlet air lines, the topmost point of the air line was located above the maximum possible liquid level in the bubble column, as shown in Figure 1. As an asphyxiant and potentially an explosive gas, H_2_S was omitted due to health and safety concerns so as to minimize risks to the personnel.

### Outlet Configuration

The liquid outlet from the bubble column was routed to a 300 mm high and 24 mm diameter gas–liquid separator. The connecting line from the bubble column outlet to the gas–liquid separator was gently sloped to prevent airlock formation and minimize fluctuations in the flow. From the gas–liquid separator, the liquid was routed through a U-tube configuration to a position higher than the bottom of the separator. This ensured that a minimum liquid level was maintained in the separator. As can be seen from Figure 1, at the higher location, one end of the liquid outlet from the gas–liquid separator was kept open to the atmosphere using a tee connection. This avoided siphonic emptying of the gas–liquid separator. The system then became self-adjusting from a liquid flow rate perspective, whereby the liquid outlet flow rate would match the liquid inlet flow. The spent algal solution was collected and disposed following safety guidelines.

The gas from the gas–liquid separator was connected to the upgraded biomethane exiting the top of the bubble column. The combined gas flow passed through a mass flow meter (Bronkhorst F101D Low-ΔP-Flow Thermal Mass Meter) to vent at a safe location, as shown in Figure 1. A gas sampling point was also added before the flow meter to measure the gas composition with minimal errors. A detailed discussion for such a setup is provided in the section titled “Startup and Design Modifications”. A differential pressure gauge was fitted to the gas outlet from the bubble column to measure the pressure in the entire setup during continuous operation.

### Process Control and Measurements

All operations were manually monitored and controlled, with the exception of the temperature controls. The inlet pH, alkalinity, and DO of the algal solution were measured and controlled as necessary, as described in the section “Experiments and Methods”. A constant pressure was maintained at the gas outlet from the bubble column during the entire duration of the experiments by adjusting the constriction of the downstream gas line tubing. The pH of the liquid at the outlet of the gas–liquid separator was monitored at regular intervals. The gas flow rate was logged continuously. When both the gas flow rate and the pH of the outlet liquid became constant simultaneously (less than 5% variation in both the gas flow rate and the pH was achieved over a period of 120 s), the gas was collected for content analysis (specifically the composition of CO_2_, N_2_, and O_2_) by gas chromatography. Subsequently, the individual volumetric flow rates of the respective gases at steady state were obtained to allow evaluation of the performance of the bubble column.

### Performance Evaluation

Performance of the bubble column reactor was evaluated based on the CO_2_ and O_2_ content of the ensuing biomethane. The obtained values were compared with the existing literature to validate the design and operations of the bubble column setup for photosynthetic biogas upgrading.

### CO_2_ Absorption

CO_2_ absorption in a bubble column is typically analyzed in terms of the absorption reaction rate and overall mass-transfer coefficient.^14^ The mean steady-state absorption rate of CO_2_ [mol/L/s] can be described by the shell material balance in terms of the molar flow [mol/s] of CO_2_ in the biogas entering the column (*N*_CO_2_,BG_), the molar flow of CO_2_ in biomethane (*N*_CO_2_,BM_), and the volume of the liquid in the column (*V*_BC_).^14^ This requires several assumptions. Ideally, the presence of microalgae in the alkaline water as a solid phase would require the current system to be evaluated as a slurry bubble column. However, even with a microalgae concentration up to 1 g-DW/L, this would represent less than 0.1% total solids in the liquid. As such, with low solid concentrations, the impact of the solid phase can be neglected^36,37^ and the overall system can be assumed to be well represented by the two-film theory.^11,38^ Additional assumptions include the following:

i. assuming a thoroughly mixed gas and liquid phase in plug flow under isothermal conditions;^14^
ii. minimal absorption of nitrogen;^14^
iii. applicability of ideal gas laws;
iv. nitrogen, carbon dioxide, and oxygen are the primary gas-phase components resulting in a ternary system; and
v. fast chemical reactions typically above pH 8.^35^

From the assumption of insolubility of nitrogen and minimal nitrogen stripping from the algal solution, the total molar flow of nitrogen must be constant at the inlet (*N*_N_2_,BG_) and exit (*N*_N_2_,BM_) of the column.^14^ Thus, *N*_CO_2_,BM_ can be expressed in terms of *N*_CO_2_,BG_, the molar fraction of CO_2_ at the inlet (*y*_CO_2_,BG_), and the molar fractions of CO_2_ and O_2_ at the exit of the bubble column (*y*_CO_2_,BM_ and *y*_O_2_,BM_ respectively), as shown in eq 5
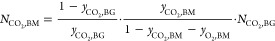
5

Consequently, the absorption rate of CO_2_,  [mol/L/s], can be written as per eq 6,^14^ which can then be expanded to eq 7 using eq 5 and  following ideal gas laws
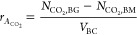
6
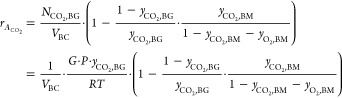
7where *G* is the biogas flow rate into the bubble column [m^3^/s], *P* is the absolute pressure of the gas phase [Pa], *R* is the universal gas constant [8.314 J/mol/K], and *T* is the temperature [K].

The overall mass-transfer coefficient  [s^–1^] for CO_2_ absorption accounting for both the gaseous side and liquid side mass-transfer coefficients was estimated via a lumped approach as shown in eq 8(11,39)
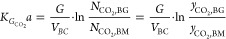
8

The CO_2_ removal efficiency (RE_CO_2_) is a widely used parameter to evaluate the performance of a bubble column for CO_2_ removal from biogas. Knowing the CO_2_ flow rates in and out of the bubble column, RE_CO_2_ [%] can thus be evaluated as per the eq 9
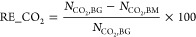
9

### O_2_ Stripping

Stripping of dissolved oxygen from the liquid phase is governed by the volumetric mass-transfer coefficient from the liquid to the gaseous phase, represented as  [s^–1^].^40^ As described by Franco-Morgado et al. (2017),^27^ the rate of the oxygen stripping  [mol/L/s] can be calculated from the mass balance at the gas–liquid interface by using the two-film theory as per eq 10
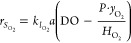
10in which DO represents the instantaneous dissolved oxygen concentration [mol/L] in the bulk liquid, *y*_O_2__ is the instantaneous O_2_ concentration in the gas flow, *P* is the absolute pressure of the gas phase, and *H*_O_2__ is the Henry’s law constant for oxygen (635 L·atm/mol at 20 °C^41^).

On the gas side, the rate of oxygen leaving the column could be given as the difference of the molar flow of oxygen entering (*N*_O_2_,BG_) and exiting (*N*_O_2_,BM_) the column per its unit volume. Considering the shell material mass balance, under steady state, as suggested by Jin et al. (2001),^42^ can thus be estimated at the entry of the bubble column PBR as per eq 11
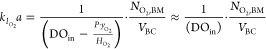
11where the term  can be neglected due to the negligible oxygen concentration in the feed biogas.

Alternatively, numerous correlations are also available to estimate . However, the results vary significantly due to the type of gas–liquid system, the column diameter, and the sparger design (especially for smaller-diameter columns and lower gas superficial velocities). As an approximation, the correlations by Shah et al. (1982)^43^ for cross nozzle spargers were considered for cross-validation of the bubble column performance. The correlation, as depicted in eq 12, describes  in terms of the superficial gas velocity, *u*_G_, as shown below
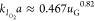
12

### pH, Alkalinity, and Carbon Mass Balance

Under steady-state operations, the CO_2_ removed from the biogas would be absorbed into the liquid as bicarbonate following eq 4 with a corresponding drop in pH. Assuming minimal algal growth within the bubble column, the carbon mass balance (in terms of molar flow rates) between the inlet and outlet of the bubble column could be written as per eq 13
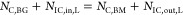
13in which *N*_C,BG_ and *N*_C,BM_ are the molar flow rates of carbon [mol/L-C] in the biogas and upgraded biomethane, respectively, while the respective molar flow rates of inorganic carbon (IC) on the liquid inlet and outlet are denoted as *N*_IC,in,L_ and *N*_IC,out,L_. Consequently, following from eq 5, the dissolved IC loading in the bubble column (ΔDIC_BC_) [mol/L-C] relative to the liquid flow rate (*L*) [m^3^/s] in the bubble column can be described by eq 14 as
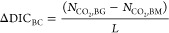
14

In photosynthetic biogas upgrading using inorganic media only for microalgae growth, the IC concentration can be interchangeably used for alkalinity.^20^ This is because at a pH above 8, the primary forms of IC in solution are [HCO_3_^–^] and [CO_3_^2–^]. Thus, the increase in the IC concentration due to the absorption of CO_2_ in the solution can be equated to the increase in total alkalinity. Correspondingly, the carbon mass balance between the entry (i) and exit (o) of the column could be re-arranged from eqs 13–15
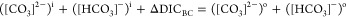
15in which the instantaneous concentrations of [HCO_3_^–^] and [CO_3_^2–^] can be determined after Kishi et al. (2019)^44^ via eqs 16 and 17, respectively, in terms of the corresponding pH and alkalinity as
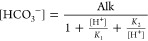
16
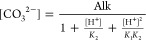
17where *K*_1_ and *K*_2_ are the stoichiometric constants for bicarbonate and carbonate, respectively; pH can be determined as −log[H^+^]; and Alk represents the alkalinity [mol/L-C]. *K*_1_ and *K*_2_ can be estimated in terms of p*K*_1_ and p*K*_2,_ by eq 18(45)
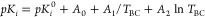
18where p*K*_*i*_ is −log_10_*K*_*i*_ and p*K*_*i*_^0^ is the dissociation constant in pure water accounting for the impact of temperature for *i* = 1,2. The values of p*K*_1_^0^ and p*K*_2_^0^ can be estimated from eqs 19 and 20 according to Millero et al. (2006).^45^ On the other hand, the values of the adjustable parameters *A*_0_, *A*_1_, and *A*_2_ accounting for the impact of salinity were directly obtained from Millero et al. (2006)^45^
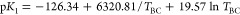
19
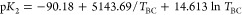
20

## Experiments and Methods

### Microorganisms and Culture Conditions

All operations and experiments were performed with non-axenic strain of *Arthrospira* (*Spirulina*) *platensis* SAG 85.79, procured from Sammlung von Algenkulturen Goettingen (SAG), Germany. The microalgae were batch-cultivated in 5 L Erlenmeyer flasks using the modified Zarrouk’s media after Madkour et al. (2012)^46^ comprising (g L^–1^ of distilled water) the following: 16.80 NaHCO_3_, 0.50 K_2_HPO_4_, 2.50 NaNO_3_, 1.00 K_2_SO_4_, 1.00 NaCl, 0.2 MgSO_4_·7H_2_O, 0.04 CaCl_2_·2H_2_O, 0.01 FeSO_4_·2H_2_O, 0.08 ethylenediaminetetraacetic acid-Na, and 1 mL of the micronutrient solution. The micronutrient solution was made up with 2.86 g/L H_3_BO_3_, 1.810 g/L MnCl_2_·4H_2_O, 0.222 g/L ZnSO_4_·7H_2_O, 0.0177 g/L Na_2_MoO_4_, and 0.079 g/L CuSO_4_·5H_2_O. All batches were inoculated with 0.15 g-dry weight (DW)/L of algae. The temperature was kept constant at 20 ± 1 °C, and illumination was programmed to provide 16:8 h of light/dark cycles using cool white fluorescent lamps at an intensity of 5–6 klux (75 ± 5 μmol m^–2^ s^–1^). The algae were continuously agitated with air to ensure adequate mixing and promotion of algae growth. The strain was maintained in 50 mL batches inside 100 mL sterilized Erlenmeyer flasks using modified Zarrouk’s medium at 20 ± 1 °C and a pH between 9 and 10 and by shaking twice daily manually. Illumination was maintained at 2.5 klux (33.75 μmol m^–2^ s^–1^) with a 16:8 light/dark period, and the cultures were transferred to a new medium every 3 weeks.

### Commissioning and Performance Evaluation of the Bubble Column Operations

In accordance with the primary objective of the work, the experimental design was aimed not only at evaluating the performance of the bubble column but also at identifying the potential challenges toward its operation and evaluation procedures. Corresponding to each challenge, design modifications were implemented with a focus on both the laboratory-scale and commercial-scale operations of such systems. For this, simplistic experiments were performed with synthetic biogas containing 40% CO_2_ and the remaining N_2_. The flow rate was kept constant at 54.83 mL_n_/min (corresponding to a *u*_G_ of 0.2 cm/s), typical of the upper limits of current experimental values. The resultant EBRT was 4.5 min, corresponding to those typically used in industrial bubble columns.^15^ On the other hand, the liquid flow rate, and hence the L/G ratio, was varied until no statistically significant change in the biomethane CO_2_ composition was noticed. Accordingly, the L/G ratio was increased in steps of 0.1 from an initial value of 0.1 (corresponding to the lower limit of the peristaltic pump operations).

In the two-step bubble column PBR configuration, the culture medium can be circulated into the bubble column from the PBR either prior to harvesting or post harvesting. All commissioning and troubleshooting was performed with a liquid of a low algal concentration of 0.05 g-DW/L, typical of those obtained post harvesting of microalgae. On successful completion of all low algal concentration trials the algae concentration increased to 0.75 g-DW/L to evaluate the effects of higher algal concentrations on the bubble column operation. The higher concentration value was in accordance with those achievable in open ponds and closed PBRs alike prior to harvesting.^47,48^ Additionally, to evaluate any potential impact of photosynthesis on biogas upgrading, experiments were performed under continuous illumination between 3 and 5 klux (40.5–67.5 μmol m^–2^ s^–1^) (similar to those used for *Spirulina* cultivation). For ease of following the paper, from this point forward, all low-algal-concentration experiments will be denoted as LON and all those with high algal concentrations will be denoted as HON.

The alkaline algal liquid for biogas upgrading was prepared by diluting 2–3 g-DW/L live *S. platensis* cultures with distilled water. The pH of the inlet algal solution was maintained at 9.35 ± 0.05 for all experiments by addition of sodium carbonate–bicarbonate wherever applicable or by purging with CO_2_ within the optimal range of *S. platensis* (pH 8 to 11).^32^ The alkalinity was controlled at 2.8 ± 1 g-IC/L by addition of sodium carbonate–bicarbonate or by diluting with distilled water and subsequent adjustment of the algal density. All bubble column experiments were performed at a temperature of 21 ± 2 °C; the DO of the algal solution varied between 7 and 10 mgO_2_/L (90 ± 10% saturation value) as per those recorded during batch cultivation of algae. Each experiment was performed in triplicate.

### Analytical Procedures

The algal density in solution was measured as the optical density using a VWR V-3000 PC manual spectrophotometer, calibrated against the microalgae DW. For this, the DW of microalgae was determined by filtering 2 mL of the sample through pre-washed and dried Whatman filter papers (47 mm diameter and nominal pore size 0.45 μm). Each filtered sample was washed thrice with distilled water to remove residual salts, dried in an oven at 80 °C for 4 h, and cooled in a desiccator to obtain the corresponding DW.^46^ λ 530 nm was selected after multiple wavelength screening to calibrate the spectrophotometer optical density to the standard microalgae DW. Both pH and DO were measured using a handheld pH meter (VWR MD 8000H Multi Parameter Meter) fitted with interchangeable pHenomenal VWR pH/ORP and pHenomenal VWR OPOX 11-3 sensors, respectively. Alkalinity was determined by titrating the sample in a Titronic Universal Titrator with 0.1 N H_2_SO_4_ up to an end point pH of 4.5 as per the basic methodology described in method no. 2320B of APHA.^49^ Salinity (parts per thousand) due to carbonate and bicarbonate salts can be estimated in terms of the amount of their respective oxides per kg of water.^50^ Assuming all other salts to be negligible due to the use of distilled water, the salinity of the medium was thus estimated from the theoretical amount of sodium oxide per liter of water. For this, the complete oxidation of added amounts of sodium carbonate and sodium bicarbonate was considered according to eq 21
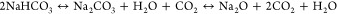
21

The upgraded biomethane flow rate was measured in terms of equivalent N_2_ flow [mL_n_-N_2_/min] using a thermal mass flow meter (Bronkhorst F101D Low-ΔP-Flow Thermal Mass Meter). The corresponding steady-state composition was measured using an Agilent 7890B Gas Chromatograph (USA) equipped with a thermal conductivity detector and a 5A column and calibrated with 2% O_2_ and 30% CO_2_ (balance N_2_) from Buse Gases Ltd, UK. Subsequently, an online gas Converter tool by Fluidat (Bronkhorst) was used to record the actual flow rates of the component gases at the bubble column outlet.

### Statistical Analysis

The spectrophotometer standard curve for measuring the algal density was estimated by fitting a linear curve via Origin 8.5 software using the root-mean-square prediction error to evaluate the accuracy of the linear fit. Statistical analysis of the performance of photosynthetic biogas upgrading was conducted using Minitab version 19 (Minitab LLC., Pennsylvania, USA). Significant differences in the results between each trial run were assessed by both one-way and two-way analysis of variance using Tukey tests for post-hoc analysis (*P* < 0.05).

## Startup and Design Modifications

### External Oxygen Ingression into Upgraded Biomethane

At the start of operations, the oxygen concentrations in the upgraded biomethane ranged between 3 and 4%. Although the values were similar to those reported in the literature,^17,29^ a feasibility check for the results was performed. For this, the  was estimated for the experimental trials as described via eq 11 and compared to the empirical value obtained through eq 12. The experimental values were at least 4.5 times that of the empirical value of 0.0029 s^–1^. A mass balance based on the assumptions discussed in the section “Performance Evaluation” was further conducted considering the oxygen in biomethane to be derived solely from the dissolved oxygen of the circulating low-algal-concentration liquid. Assuming the dissolved oxygen in the inlet liquid to be 10 mg/L and that it was completely stripped from the liquid by biomethane, a theoretical maximum oxygen content in biomethane was obtained. However, the observed oxygen concentrations in the upgraded biomethane were at least 10 times that of the theoretical maximum. This indicated significant oxygen ingression in the system, requiring substantial design modifications.

To understand the source of oxygen ingression, the oxygen concentration was measured at different locations of the experimental setup with only nitrogen gas flowing through the system. From the results, the high permeability of the silicone tubing used in the system was recognized as the major factor affecting O_2_ concentrations in the upgraded biomethane, followed by the use of gas bags for sampling biomethane. However, industrial settings operating with impermeable steel pipelines would seldom face gas ingression issues. Thus, to enable the accurate measurement and replication of industrial operations in the current experimental setup, the following modifications in system design were implemented. The results before and after the modification are discussed in the Results section.

I. All gas line tubing was replaced with the Tygon 3603 tubing having at least 50 times less permeability than silicone-based rubber tubes.
II. The gas bag was replaced with a 10 mL plastic syringe for gas sampling.
III. The gas collection point was shifted before the flow meter to minimize the measurement error.
IV. A much smaller outlet residual oxygen flow was still observed when nitrogen gas was flowing through the system with the modified tubing. To further minimize this error, the residual flow of oxygen in the gas tubing was subtracted from the oxygen flow rates in the raw biomethane. Therefore, a two-point oxygen and nitrogen correction was applied to increase the measurement accuracy. For this, the outlet gas was measured for its composition and flow rate with flowing nitrogen at 25 and 50 mL_n_/min into the system, yielding an oxygen and nitrogen correction factor. These correction factors were then subtracted from the respective gas flow rates in the upgraded biomethane for each experimental run to evaluate the corrected oxygen and nitrogen concentration in biomethane.

### Condensation

Moisture condensation buildup within the gas tubing at the bubble column outlet was observed over time affecting the accuracy of measurements. Enhanced mass transfer to achieve higher CO_2_ removal, also resulting in enhanced evaporation,^51^ caused considerable humidification of biomethane. The issue was solved by immersing a length of a large-diameter tube in an ice bath to act as the condensate trap, as can be seen from Figure 2e. In an industrial setup, a condenser would thus be necessary to remove moisture from the upgraded biomethane to the required limit of downstream operation.

### Foaming

**Figure 3:**
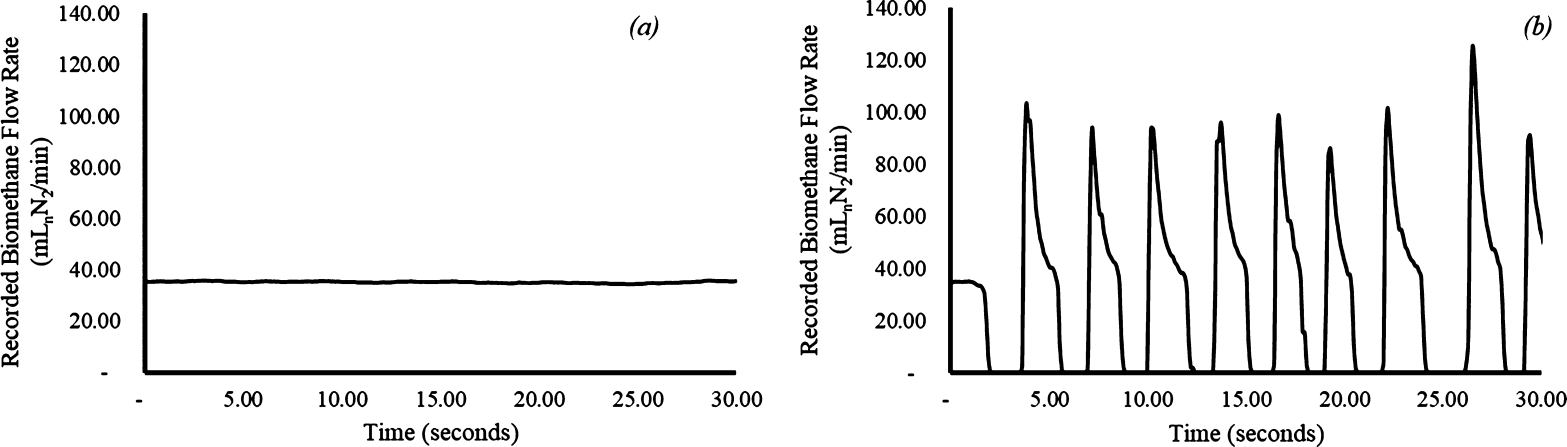
Recorded biomethane mass flow profiles at a similar L/G ratio (0.3) (a) without (low algal concentrations) and (b) with foaming (high algal concentrations).

Significant foaming was observed (Figure 2c) above the liquid column, especially during operation with higher algal concentrations similar to those reported by Besagni and Inzoli (2017),^52^ while working with active organic compounds and aspect ratios greater than 10. Significant foaming properties of *S. platensis* recently documented in detail by Buchmann et al. (2019)^53^ further confirm the observed phenomenon. Over time, the foam rose to the top of both the bubble column and gas–liquid separator, coating the walls of the gas tubes (Figure 2c) and causing significant spikes in gas flow (Figure 3b). A 500 mL foam trap added between the bubble column and the gas flow meter prevented any foam overflow into the gas flow meter, although the oscillations in the gas flow readings remained. In addition, large foaming incidents increased the pressure in the system, causing the gas to flow out through the liquid outlet in the gas–liquid separator, especially at higher liquid flow rates. Therefore, in industrial-scale bubble columns, mechanical foam breakers or the addition of anti-foam agents based on their suitability of application could aid operations with a high algae concentration.

### Clogging

Operation with a higher algal concentration increased clogging in the U-bend at the outlet of the gas–liquid separator. This restricted outlet flow of the circulating liquid, causing the liquid in the bubble column to rise above its liquid outlet level. This in turn increased the pressure inside the bubble column due to the reduction in the gas headspace. The problem was solved by increasing the tubing diameter at the liquid outlet of the gas–liquid separator. In an industrial setting without an outflow pump, the liquid outlet pipe diameter could therefore be a critical design challenge.

### Stability of Bubble Column Operation

**Figure 4:**
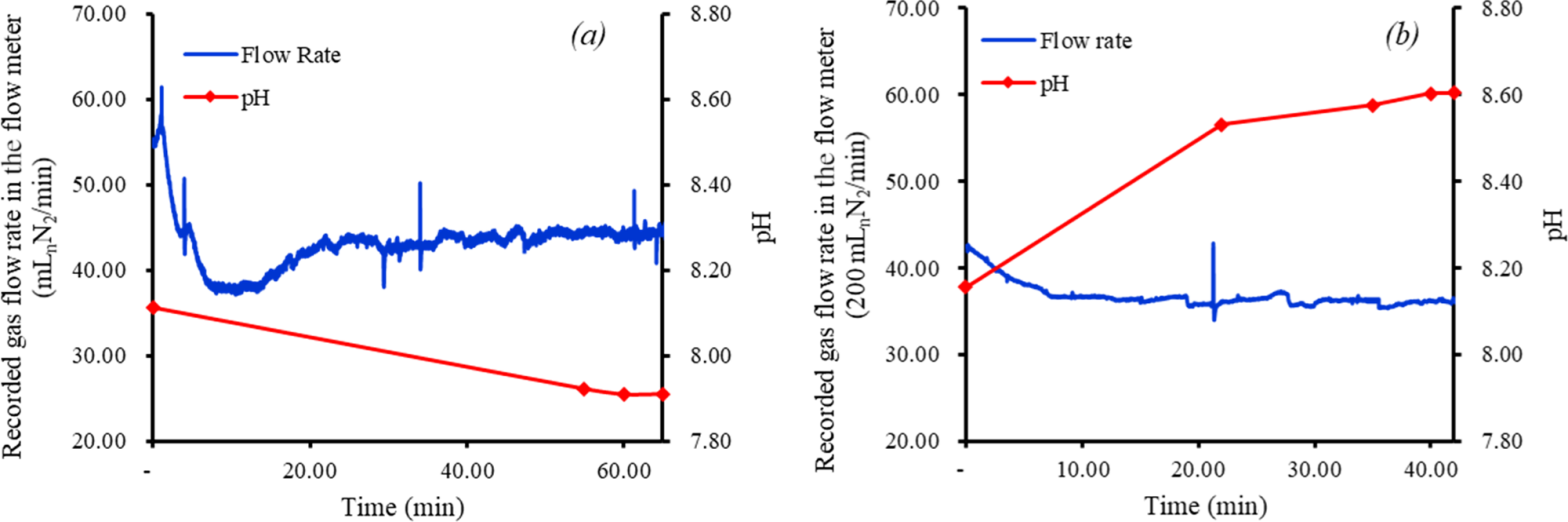
Variation in outlet biomethane flow and liquid pH before reaching the steady state. The examples chosen are (a) L/G 0.1 and (b) L/G 0.3 for experiment LON (low algal concentration). It is to be noted that the initial pH at the outlet of the bubble column for (a) was 8.113, while for (b), it was 8.16 from the respective preceding experiments. The data are from the pre-modified setup, chosen for better representation.

All measurements were performed at steady state, characterized by both a constant gas outflow rate and a constant pH of the outlet liquid. To achieve steady-state operations, both stability of the volumetric flow rate and pH were considered necessary and sufficient. As illustrated in Figure 4, the time required to reach the steady state was around 40 min for an L/G ratio of 0.4, governed by the stability of the pH. It is in close approximation to Chen et al. (2015),^14^ who also obtained a stability time of 40 min for CO_2_ removal with NaOH. However, the time to reach the steady state was much longer for a lower L/G ratio of 0.1 (Figure 4a). Thus, irrespective of a steady state being attained before the indicated times, to increase robustness in the data obtained, a minimum time of 40 min was used for each run based on the approximation of gas line length, the gas flow rates, and literature suggestion from Chen et al. (2015).^14^

## Results

All initial trials were performed with a low algal concentration (0.05 g-DW/L) to establish a working bubble column setup for biogas upgrading with microalgae. For every assessment, therefore, the impact of the algal presence was neglected unless explicitly mentioned.

### Impact of Design Changes on System Performance

Comparable pH at the liquid outlet both before and after the changes to the gas tubing and gas sampling arrangements indicated a similar performance of the bubble column in both instances. However, as can be seen from Table 2, both CO_2_ and O_2_ in the upgraded biomethane changed significantly after the modifications. The higher CO_2_ content recorded after implementing the respective design changes could be explained by the fact that due to the permeability of silicone tubing, CO_2_ was lost through diffusion. The effect became more pronounced for trials with higher CO_2_ flow rates in the ensuing biomethane, making the selection of tubing a critical aspect in the design of such a laboratory-scale setup. It is to be understood here that unlike the variation in the oxygen content in biomethane, the variations in the result (in terms of percentage composition of biomethane) from the loss of CO_2_ would be much less pronounced due to its higher flow rates. This is especially true for biomethane with a low CO_2_ content; due to the much lower atmospheric CO_2_ concentrations, the CO_2_ efflux would be minimal. Thus, a CO_2_ correction step was omitted without the loss of generality.

**Table 2:** Effect of Design Changes on the Mass Flow Rates of CO_2_, O_2_, and N_2_ Contained in Upgraded Biomethane in Comparison to the Inlet Flows for Operation under Low Algal Concentrations, Indicating the Adequacy of the Design Changes and Measurement Techniques Adopted During Commissioning the Bubble Column Setup^a^

								outlet biomethane flow (mg/min)					
	inlet biogas flow (mg/min)							initial setup			modified and commissioned setup		
L/G ratio	CO_2_	N_2_	O_2_	inlet liquid flow (mL_n_/min)	maximum inlet DO (mg/L)	O_2_ flow in the inlet liquid (mg/min)	pH of the outlet liquid	CO_2_	N_2_	O_2_	CO_2_	N_2_	O_2_
0.1	40.12 ± 0.20	38.30 ± 0.19	0.0 ± 0.0	5.48	10	0.055	7.84	10.43 ± 0.90	39.29 ± 0.26	1.66 ± 0.01	22.02 ± 0.91	38.40 ± 0.31	0.031 ± 0.027
0.2				10.97		0.110	8.06	4.09 ± 0.38	38.48 ± 0.03	1.59 ± 0.11	8.43 ± 0.33	38.33 ± 0.31	0.126 ± 0.040
0.3				16.45		0.164	8.47	1.61 ± 0.35	38.12 ± 0.09	1.65 ± 0.05	2.57 ± 0.26	38.34 ± 0.45	0.135 ± 0.067
0.4				21.93		0.219	8.74	0.8 ± 0.16	37.70 ± 0.40	1.68 ± 0.11	1.14 ± 0.13	38.33 ± 0.43	0.207 ± 0.013
0.5				27.42		0.274	8.92	0.42 ± 0.42	37.73 ± 0.43	1.60 ± 0.05	0.71 ± 0.07	38.30 ± 0.61	0.230 ± 0.040

a The molar masses of CO_2_, N_2_, and O_2_ assumed were 44, 28, and 32 g/mol, respectively.

The biggest change was observed in the oxygen content in the upgraded biomethane. From the mass balance, as can be seen from Table 2, the modified setup provided agreeable results to the theoretical maximum (corresponding to the inlet O_2_ flow rate in the liquid [mg/min] indicated in Table 2). The corresponding calculated  values ranged between 0.0013 s^–1^ for an L/G ratio of 0.1 and 0.0027 s^–1^ for an L/G ratio of 0.5. These values compared much better to both the empirical estimation [0.0029 s^–1^ from Shah et al. (1982)^43^ and the experimental values obtained in a similar bubble column by Franco-Morgado et al. 2017 (0.0016 s^–1^).^27^ Indeed, beyond an L/G ratio of 0.4, where the CO_2_ content was below 2.5%, this resulted in achieving grid quality biomethane. The resulting corrected nitrogen flow rate in the exiting biomethane of 38.33 ± 0.43 mg/min also indicated the adequacy of the methodology applied (the N_2_ inflow set at 32.9 mL_n_/min or 38.30 mg/min). All subsequent results depicted are for the modified system setup.

### CO_2_ Removal Efficiency

**Figure 5:**
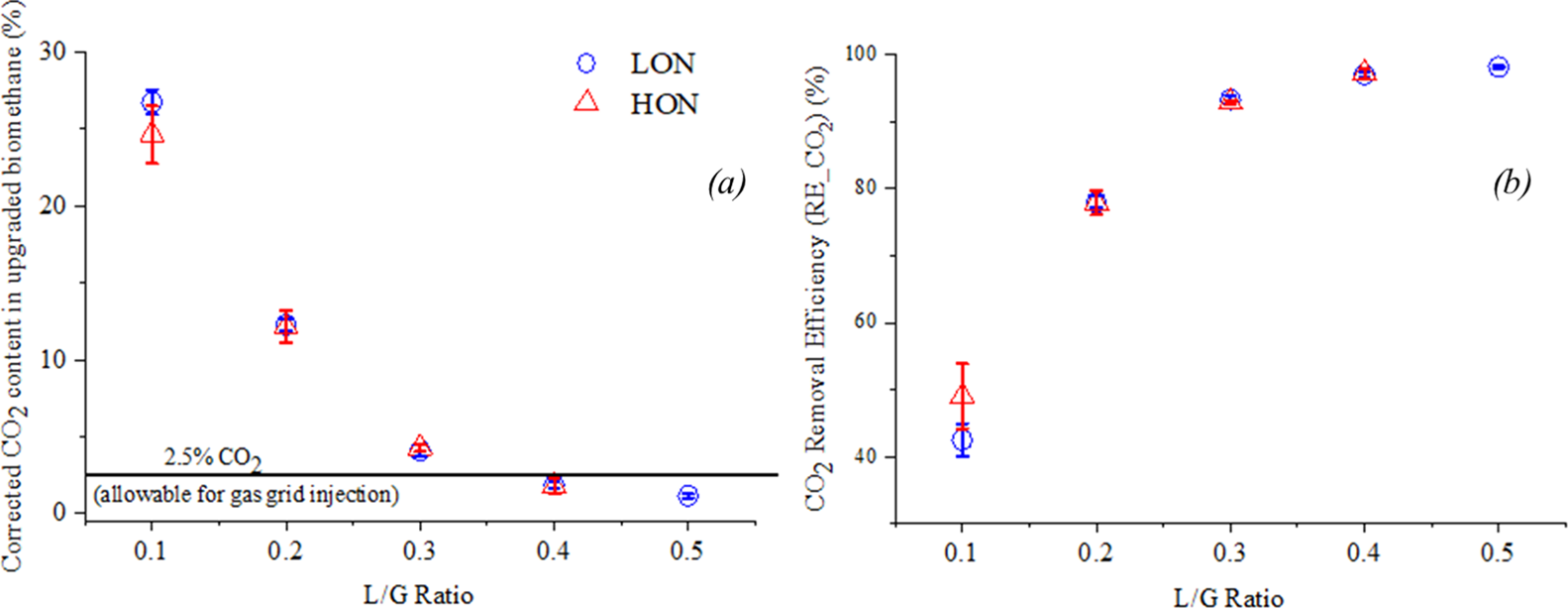
CO_2_ content (a) and removal efficiency (b) of the bubble column operated under different L/G ratios at higher and lower algal concentrations; LON: low concentration with lights on; HON: high concentration with lights on.

The variations in CO_2_ content in the outlet biomethane at different L/G ratios under both the lower and higher algal concentrations are shown in Figure 5a. For the higher concentration, an L/G ratio of 0.5 was not recorded as the experiments were deemed to provide non-conclusive evidence due to excessive foaming. A CO_2_ concentration of around 25% was observed for all runs at the lowest L/G ratio of 0.1. A rapid drop in CO_2_ content could be seen between L/G ratios of 0.2 and 0.3. Above an L/G ratio of 0.4, CO_2_ concentration levels suitable for grid injection (less than 2.5%) were consistently achieved. Indeed, between L/G ratios of 0.4 and 0.5, no statistically significant decrease in the CO_2_ content of the upgraded biomethane was observed. Statistical assessments also established that no significance of the algae concentration and the photosynthetic effect was exerted on the performance of CO_2_ removal. A study of the corresponding liquid outlet pH from Table 2 provides further insight into the system performance. At an L/G ratio below 0.1, when the CO_2_ removal was the lowest, the pH fell below 8. Subsequently, a steady increase in pH can be observed, with the highest pH recorded being 8.92 ± 0.02 at an L/G ratio of 0.5. Similar observations were made by Rodero et al. (2018),^20^ who achieved a minimal drop in pH at an alkalinity above 1000 mg-IC/L and an L/G ratio of 0.5 with sufficient CO_2_ removal.

In congruence with the CO_2_ content in the upgraded biomethane, the CO_2_ removal efficiencies (RE_CO_2_) were found to vary from 42.5 ± 2.41% (L/G 0.1) to 98.15 ± 0.22% (L/G 0.5), as plotted in Figure 5b. In comparison to Toledo-Cervantes et al. (2017),^13^ who achieved a CO_2_ removal efficiency of 70 ± 1% at an L/G ratio of 0.3, the present experiments yielded a higher RE_CO_2_ of 93.29 ± 0.66%. This could be due to the higher IC concentration in the algal solution chosen in the present study. However, for an L/G ratio of 0.5, the present results were replicable with both Toledo-Cervantes et al. (2017)^13^ and Rodero et al. (2018),^18^ who reported the corresponding values of 97.3 ± 0.1 and 97.8 ± 0.8%, respectively. Thus, for higher L/G ratios, the significance of alkalinity could be understood to be diminished, with pH being the primary factor determining the efficiency of CO_2_ removal.

### Rate of CO_2_ Absorption

**Figure 6:**
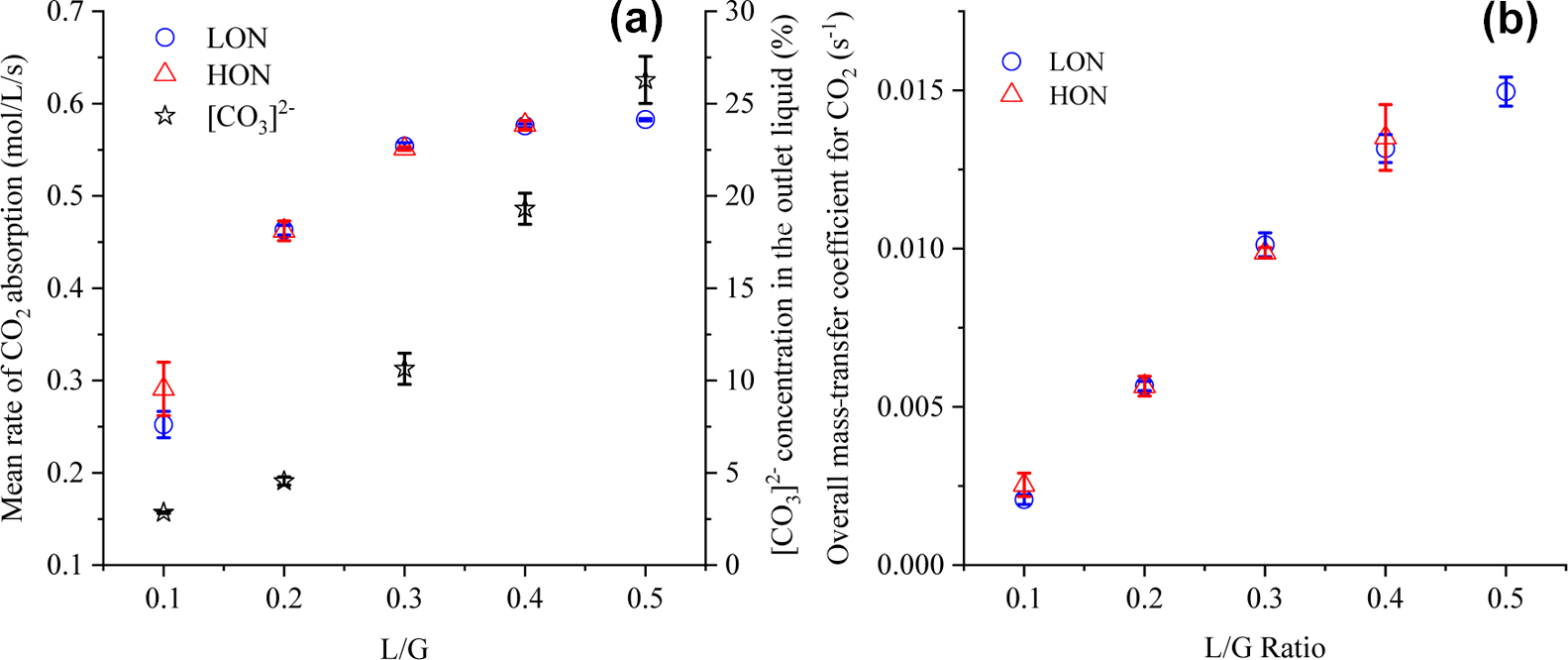
Effect of L/G ratio and algal concentration on the (a) absorption rate of CO_2_ alongside mean carbonate concentration in solution and (b) overall mass-transfer coefficient .

The mean CO_2_ absorption rates along with the carbonate concentration in the liquid at the bubble column outlet are shown in Figure 6a. The values presented are for the operations with both higher and lower algal concentrations. Considering the inlet carbonate concentration to be 48.73 ± 0.56% (corresponding to a pH of 9.35 ± 0.05 and a salinity of 10.19 ± 0.36), no statistically significant improvement in the CO_2_ absorption rates was noticed (*P* > 0.05) when the outlet carbonate concentration was above 20% (corresponding to a pH of 8.75 ± 0.02). On the contrary, a drastic reduction in the CO_2_ removal rates was observed as the carbonate concentration fell below 5% (below L/G 0.2). This conclusion matches well with the results obtained by Knuutila et al. (2010),^34^ who reported a drop in the reaction rates of over 10 times as the carbonate concentration decreased from 20 to 5%. Thus, there is a requirement for the average carbonate concentration in the liquid to be around 20% to ensure sufficient CO_2_ removal. The corresponding CO_2_ absorption rates for LON were 0.576 ± 0.002 × 10^–4^ and 0.583 ± 0.001 × 10^–4^ mol/L/s (∼14.15 × 10^–6^ mol/s normalized by a liquid volume of 0.244 L) at L/G ratios of 0.4 and 0.5, respectively. The absorption rates for LON compare well to the results of Chen and Lin (2015),^11^ who reported a CO_2_ absorption rate of 1.03 × 10^–4^ mol/L/s at 25 °C and an initial pH of 10 for CO_2_ removal in a bubble column by sodium hydroxide. The lower rates could be explained by both a lower temperature and a lower pH used in the present experiments. The comparison can be supported by the fact that irrespective of the use of Na_2_CO_3_ or NaOH, the local reaction is driven by [OH]^-^ as per eq 2.^34,35^ Indeed, below a pH of 8, due to the minimal presence of carbonates in solution, eq 4 is replaced with the following carbonic acid formation reaction (eq 22).^35^ Thus, the absorption rates would drop rapidly, and the present assumptions would not be valid to determine the kinetics of the system.^35^ No significant change in CO_2_ absorption rates was obtained when the algal concentration increased. This indicated that no impact of the algae was exerted on CO_2_ absorption, the latter being primarily driven by the [OH]^−^ ions in solution^a^^54^
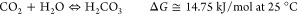
22

The overall mass-transfer coefficient, , was found to increase linearly from 0.0025 s^–1^ at L/G 0.1 to 0.015 at L/G 0.5, as shown in Figure 6b. An enhanced mass-transfer coefficient at higher L/G ratios has been previously noted as well.^33^ Indeed, although at lower L/G ratios the mass-transfer coefficient is significantly lower than that reported in the literature, for the trials with appreciable CO_2_ removal rates (L/G 0.4 and 0.5), the results match well to that of Chen et al. (2015),^14^ who reported a  of 0.015 s^–1^ at an inlet pH of 10 and 25 °C. Similar to the absorption rates, no statistically significant influence of a higher algal content can be seen from the data presented in Figure 6b.

### O_2_ Stripping

**Figure 7:**
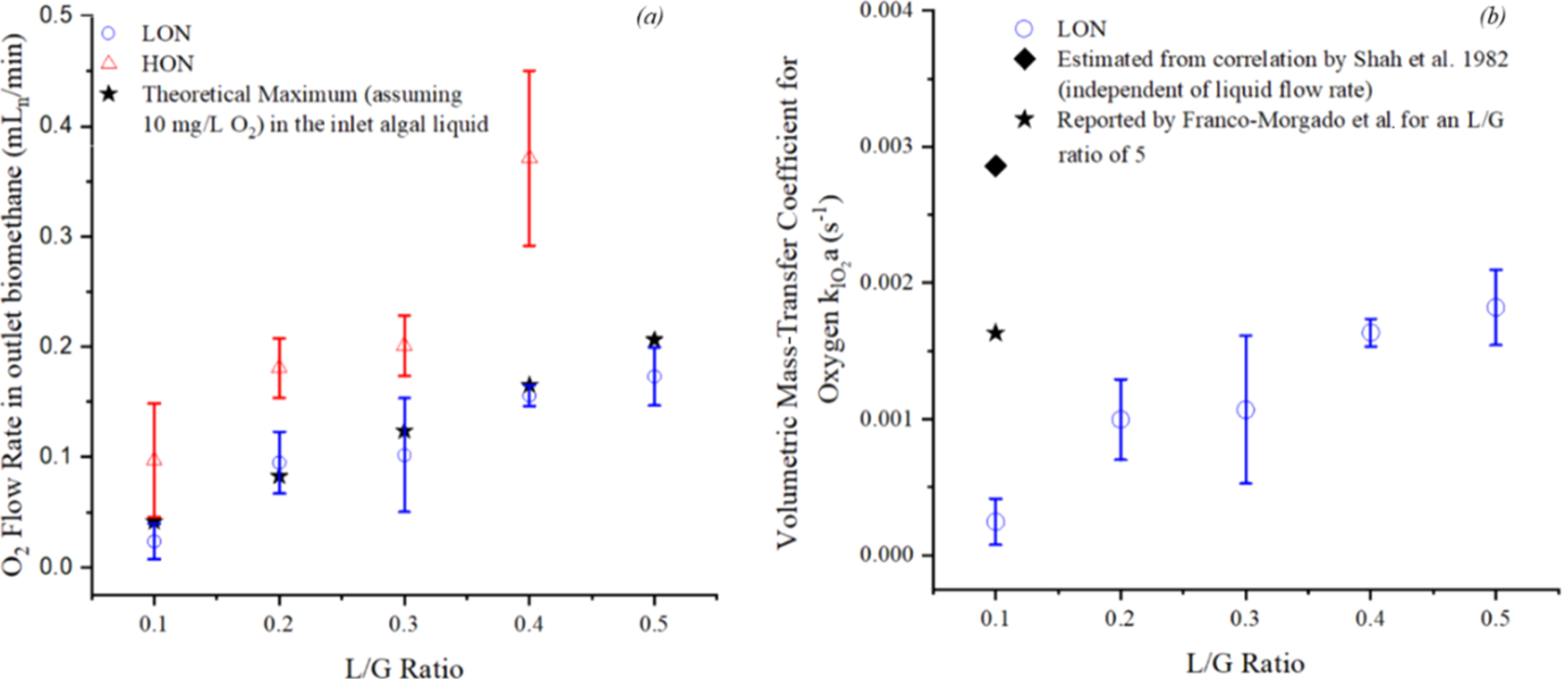
Effect of the L/G ratio and the algal concentration on (a) theoretical and measured oxygen outflow rates [mL_n_/min] and (b) overall mass-transfer coefficient for oxygen  [s^–1^]. It is to be noted that  for HON have not been evaluated as the results are confounded with the photosynthetic activity of the algae and would not depict the actual hydrodynamic performance of the bubble column.

As can be seen from Figure 7a, as the L/G ratio increased from 0.1 to 0.5, the oxygen flow rates increased from 0.023 ± 0.016 to 0.173 ± 0.026 mL_n_/min, respectively, for the operations with a low algae concentration following the trend of the theoretical values. However, the corresponding oxygen flow rates while operating with a higher algal concentration not only were much higher but also showed higher variability. One possible explanation could be the non-uniform mixing because of the filamentous microalgae in liquid solution. Similar observations of improved mass transfer due to an increased algal concentration in the liquid phase have been previously reported by Manjrekar et al. (2017).^22^ However, to explain the flow rates above the theoretical values, it can be speculated that this increase is from the high concentration of photosynthesizing microalgae present in the system.

To assess the hydrodynamic performance of the bubble column, the volumetric mass-transfer coefficient is presented in Figure 7b and compared with the literature. In accordance with the above discussion, the  was not calculated for operations with higher algal volumes as there was a significant probability of confounding the results with the oxygen produced via photosynthesis. As can be seen, thus, there is good agreement between the predicted (0.0029 s^–1^), literature (0.0016 s^–1^), and experimental results (varying between 0.0002 and 0.0018 s^–1^ at L/G ratios between 0.1 and 0.5, respectively). These results further compare well to multiple literature sources using a similar column diameter and *u*_G_, whereby a  of less than 0.01 s^–1^ can be seen, irrespective of operating conditions such as pH.^55,56^ Nonetheless, the present results also indicate that the liquid flow rate would exert a relatively large influence on the volumetric mass-transfer coefficient of oxygen. This is unlike most correlations for estimating  that neglect the impact of the liquid velocity. As explained by Besagni et al. (2018),^57^ this occurs when the liquid and gas flow rates become comparable, which indeed is the case for the present operations. An increase in  has also been reported by Chaumat et al. (2007)^58^ influenced by an increased liquid flow rate, although the gas superficial velocity is still the most significant factor affecting the same. Therefore, in the bubble column for photosynthetic biogas upgrading, the liquid flow rates would play a definitive role in determining the hydrodynamic performance of the bubble column, especially with relation to oxygen stripping. This is supported by multiple literature sources that have claimed L/G ratio to be a significant factor in controlling the oxygen concentration in the upgraded biomethane.^16,28^

**Figure 8:**
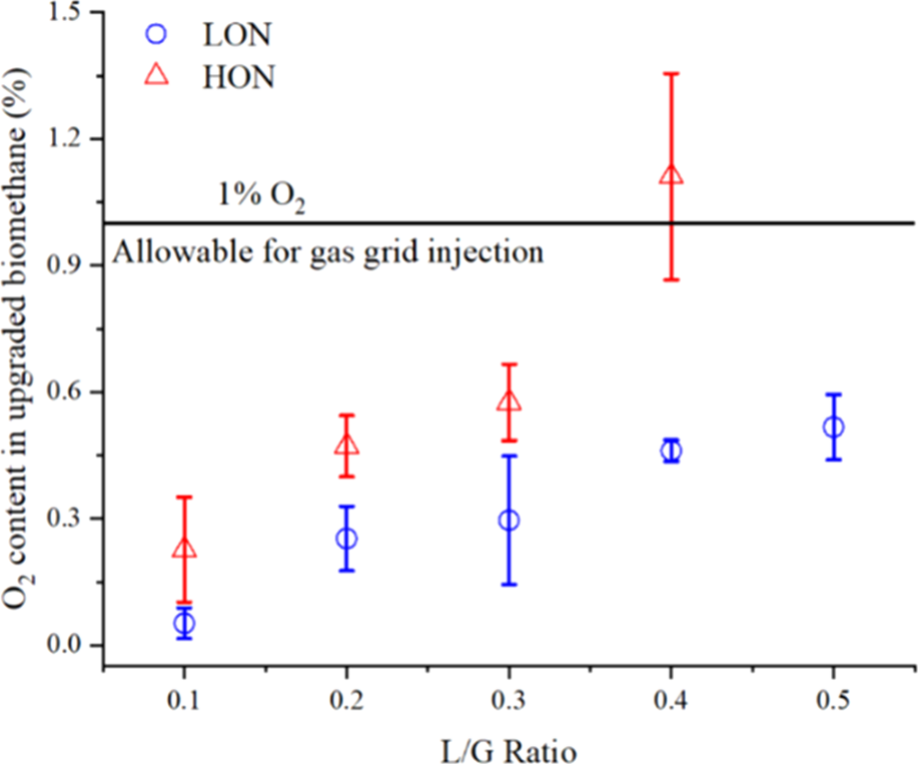
Oxygen concentration in the upgraded biomethane under different operating conditions in the lab-scale bubble column.

The corrected oxygen concentrations in the upgraded biomethane are depicted in Figure 8. During operation with a lower algae concentration, the O_2_ content in the upgraded biomethane increased from 0.05 ± 0.04% at an L/G ratio of 0.1 to 0.52 ± 0.08% at an L/G of 0.5. In addition to increased oxygen outflow, a higher CO_2_ removal efficiency was also instrumental in increasing the oxygen content in the upgraded biomethane with the increase in L/G ratios. From the perspective of O_2_ content in the biogas, the results match well with those in the literature while working with a lower algal concentration post harvesting. For example, Toledo-Cervantes et al. (2017)^16^ reported an O_2_ concentration of 0.1% ± 0.0% at an L/G ratio of 0.5 and a pH of 10.2 ± 0.2 at the bubble column inlet. The low values were mostly due to the consumption of dissolved oxygen by H_2_S in biogas. Similarly, Rodero et al. (2019)^24^ also obtained an O_2_ concentration around 0.2% for an L/G ratio between 0.3 and 0.5 and pH 10 while operating with biogas containing 300 ppm H_2_S. On the other hand, Marín et al. (2018)^17^ found 0 to 3.4% O_2_ variation in the upgraded biogas at an L/G of 1 and the pH varying between 9.3 and 9.7.

In the present work, a less than 1% O_2_ concentration has been achieved while working with low-concentration algal liquids without H_2_S in inlet biogas. This signifies the possibility to achieve grid quality oxygen limits in photosynthetic biogas upgrading even without requiring H_2_S from biogas for oxygen removal. Combined with CO_2_ concentrations below 2.5% at L/G ratios of 0.4 and 0.5, the ensuing biomethane in the present form would thus be suitable for gas grid injection.

The corresponding oxygen contents in the upgraded biomethane for operation with higher algal concentrations were 0.23 ± 0.12% for an L/G of 0.1 and 1.11 ± 0.24% at an L/G of 0.4. The higher oxygen flows from enhanced mixing and the presence of photosynthesizing microorganisms would therefore render biomethane unsuitable for gas grid injection above an L/G ratio of 0.4 from the perspective of O_2_. The operations with higher algal concentrations would therefore be detrimental to achieving grid quality biomethane and hence the applicability of the photosynthetic biogas upgrading system.

### Carbon Balance

**Figure 9:**
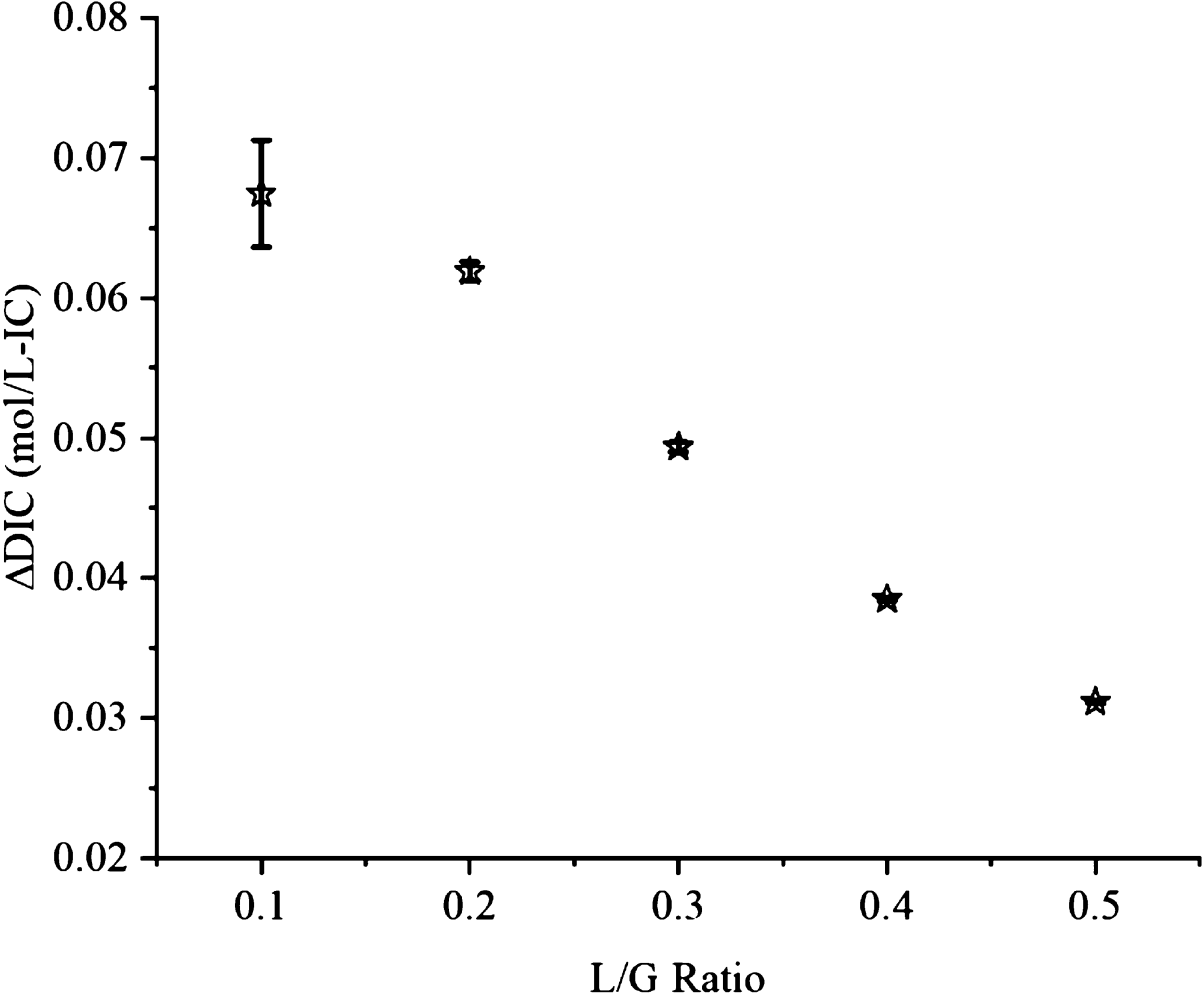
Variation in the rate of carbon addition with respect to the L/G ratio.

The effective IC addition into the liquid medium from CO_2_ absorption, as can be derived from eq 14, is shown in Figure 9. With the increase in the L/G ratio, signifying an increase in the liquid flow rate, the CO_2_ absorption rate increases as well. However, the increased liquid flow rate results in a lesser quantity of CO_2_ being absorbed per unit liquid flow rate. Thus, the DIC addition to the algal solution would decrease as the L/G ratio increases. As such, the carbon assimilation by bicarbonate formation in the circulating liquid drops from 0.067 ± 0.004 mol/L at L/G 0.1 to 0.031 mol/L at L/G 0.5. For microalgae subsequently grown using the inorganic nutrient medium, this reduction of carbon addition rate is thus of considerable significance for further integration and optimization of the overall photosynthetic biogas upgrading technology.

## Discussion

### Validation and Scale-up Perspective of the Bubble Column Setup

The results from the initial experiments indicate both the accuracy and reliability of obtained measurements and how well the designed bubble column configuration compares with those in previously published research studies, hence validating the designed lab-scale setup. The performance of the bubble column matches well to previous lab-scale experiments for photosynthetic biogas upgrading, in which an L/G ratio of 0.5 provided sufficient removal of CO_2_ for grid injection of biomethane.^16,20^ From the fundamental aspect of CO_2_ mass transfer and absorption rates, the corrected results after necessary system modifications show close proximity to previously published research.^14^ As for the bubble column hydrodynamics, both the overall CO_2_ mass-transfer coefficient  and the volumetric liquid side O_2_ mass-transfer coefficient  agree well with the existing literature at a similar scale of the bubble column and similar superficial gas velocities.^14,59^ The differences obtained could be argued from the variation of column diameter, comparable liquid and gas flow rates, and the sparger design, the latter being shown to noticeably impact the bubble column performance, especially at low superficial gas velocities (<0.15 m/s).^60^ It must be mentioned here that due to the use of nitrogen as a substitute for methane, nitrogen stripping from the liquid was not observed in the present experiments. However, as is evident from the literature, a similar trend exists between N_2_ and O_2_ stripping, resulting in comparable percentages of the respective gases in the upgraded biomethane.^17,23,25^ For this, it can be speculated that a similar N_2_ concentration (∼0.5%) can be envisaged in the upgraded biomethane under similar operating conditions with actual biogas.

Beyond validating the design and operations of the lab-scale bubble column for photosynthetic biogas upgrading, the corresponding assessment for scale-up is crucial from an industrial perspective. Most often, similarity in global hydrodynamic parameters, such as the gas holdup (signifying the ratio of the gassed volume of the bubble column with respect to the liquid volume prior to any gas flow), is used to predict dynamic similarities in bubble columns across the scale.^19^ For operations in the homogeneous regime, characterized by a uniform radial profile of gas holdup and limited interactions between bubbles, the similarity between gas holdup is necessary and sufficient to achieve dynamic similarity upon scale-up.^61^ In the current experiments, no observable change in the liquid column height was noticed before and after initiating gas flows in the bubble column, indicating minimal gas holdup. This is consistent with the observations by Majumder (2016),^62^ who reported a nominal gas holdup at low superficial gas velocities. Mathematically, a theoretical estimate of the gas holdup, Δ_g_, was obtained to be 0.006 via eq 22, as given by Chisti (1989)^63^ for bubble column bioreactors operating at *u*_G_ < 0.05 m/s
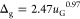
23

Improvements in sparger designs to decrease the bubble diameter and hence increase the gas–liquid contact could result in higher oxygen stripping. However, for such low gas holdup, scale-up of the bubble column reactor with minimal errors for similar sparger designs can be envisaged.^61^ Indeed, for higher superficial gas velocities, especially beyond 0.01 m/s, the gas holdup would become significant (0.03 according to eq 22). In such a situation, the Wilkinson criteria (a minimum 15 cm column diameter with a minimum aspect ratio of 5 and with a sparger pore diameter greater than 1–2 mm) would be essential for scale-up.^64,65^

In addition, below 0.01 m/s, a minimal influence of pressure on the gas holdup of the bubble column irrespective of the superficial liquid velocity has been reported.^64^ Being a necessary and sufficient condition to allow scale-up of a bubble column operation in the homogeneous regime,^61^ a similar gas holdup would therefore allow the results to be upscaled with negligible influence of the increased hydraulic pressure of a tall bubble column below 0.01 m/s. Accordingly, the present bubble column would remain scalable for biogas flow rates of up to 16.3 L/h or 271.43 mL_n_/min, corresponding to a superficial gas velocity of 0.01 m/s.

From the practicality of scale-up of the bubble column, operating under atmospheric conditions presents significant challenges. Usual operating conditions of high temperature and pressure would not be suitable while using microalgae. Although grid quality biomethane has been achieved, the lower absorption rates of around 0.58 × 10^–4^ mol/L/s would thus require a bubble column with higher aspect ratios (the present aspect ratio of 22.5 is significantly higher than aspect ratios of 3–10 usually used in the industry^66^). This requirement of large column heights would not only limit the industrial scale-up of the bubble column but also result in an increased level of oxygen stripping into the upgraded biomethane. Thus, improvement in the absorption rates of CO_2_ within the permissible limits of photosynthetic biogas upgrading via modification in temperature, alkalinity, pH, L/G ratios, and gas flow rates should be studied in detail.

### Impact of Algal Concentration and Photosynthetic Activity

The external carbonic anhydrase enzyme of *S. platensis* has been previously speculated to act as a biocatalyst for CO_2_ absorption in carbonate solution.^8^ However, the current experiments revealed no significant impact of the algal density and the corresponding photosynthetic activity on the CO_2_ removal performance of the bubble column. This could be because of the low activity of *S. platensis* at 20 °C,^32^ in which case, experiments at higher temperatures (30–35 °C) must be performed to establish a definite understanding. However, the presence of microalgae significantly increased foaming and clogging, while also increasing the oxygen flow rates within the ensuing biomethane. Thus, unless a significant benefit can be established toward CO_2_ removal from the higher presence of algae in the liquid phase, operations with a lower algal density appear preferable.

### Integration with Microalgae Cultivation

A continuous operation of the PBR-bubble column system without accumulation or depletion of carbon would require maintenance of carbon balance throughout the carbonate–bicarbonate cycle. Therefore, for operations with no organic carbon, the IC added (ΔDIC) during biogas upgrading should account for the carbon uptake by the microalgae in the PBR. As was seen from the experimental trials, an L/G ratio of 0.5 would be suitable for sufficient CO_2_ removal. Under these operating conditions, as can be seen from Figure 9, the corresponding DIC addition to the circulating algal liquid would be 0.36 g-IC/L (∼0.31 mol/L). On the other hand, the productivity of *S. platensis* at 20 °C is about 0.125 g/L/day, averaged over 15 days of cultivation.^32^ Assuming that the typical carbon content in *S. platensis* is around 50%,^67^ this therefore signifies an average carbon uptake of 0.063 g-IC/L/day (∼0.005 mol/L/day) by *S. platensis*. This indicates that the maximum hydraulic retention time of 6 days would be possible and the resulting *Spirulina* concentration would not be higher than 0.75 g-DW/L. On the other hand, for a lower L/G ratio, although the CO_2_ removal is much less, the addition of 0.804 g-IC/L would allow a maximum possible *S. platensis* concentration of 1.6 g-DW/L. The hydraulic retention time could be much higher, around 13 days. Therefore, to achieve grid quality biomethane, either the hydraulic retention time or the productivity has to be compromised unless the L/G ratio can be lowered without decreasing the biomethane quality.

### Oxygen Stripping and H_2_S

H_2_S was not included in the synthetic biogas in the present study due to health and safety regulations. However, as discussed in the Introduction section, it is well established that for conditions enabling sufficient CO_2_ removal (above pH 8.5), an appreciable H_2_S removal by chemical absorption could be simultaneously achieved with CO_2_ removal during photosynthetic biogas upgrading. Under these conditions, the effect of H_2_S on CO_2_ absorption and the bubble column performance would be negligible.^3^ Indeed, in the present study, for L/G ratios of 0.4 and 0.5, which resulted in the liquid pH to remain above 8.5 and the CO_2_ content in biomethane to be below 2.5%, the results could be considered to remain valid even with the presence of H_2_S. The absorbed H_2_S in the solution (as HS^–^) at these operating pHs would then react with DO, forming sulfates according to eq 24.^3^
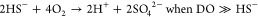
24

Using the L/G ratio of 0.5 corresponding to sufficient CO_2_ removal, a DO concentration in the feed liquid of 10 mgO_2_/L, and 500 ppm H_2_S in the feed biogas, the DO/HS^–^ ratio would be greater than 7. In this case, almost 29% of DO could be removed as sulfates, lowering the oxygen stripping into biomethane further. Similar reduction in oxygen content in the upgraded biomethane has been obtained by Bahr et al. (2014)^15^ upon increasing the H_2_S content in the inlet biogas.

## Conclusions

The present research describes the design and commissioning of a small lab-scale bubble column reactor to upgrade biogas using *S. platensis* algal solution. The setup would be able to treat up to 16.3 L/h of biogas for scalable results. The primary focus in the present work is given on its design and commissioning rather than optimization. Selection of tubing and sampling arrangements were found to significantly affect the results and system performance, especially with regard to a large oxygen ingress in the upgraded biomethane. Modification to system design, together with the implementation of correction factors to account for residual oxygen and nitrogen, resulted in achieving grid quality biomethane (CO_2_ below 2% and O_2_ below 0.55% by volume) above L/G ratios of 0.4 and low algal concentrations. Under these conditions, the corresponding CO_2_ removal efficiency achieved was above 98%. The volumetric oxygen mass-transfer coefficient  varied between 0.0002 and 0.0018 s^–1^ at L/G ratios 0.1 and 0.5, respectively, for lower concentrations of algae in the liquid phase. These results not only validated the performance of the bubble column when compared to the literature but also indicated the adequacy of the measurement and data acquisition techniques employed in the current setup. No significant impact of the algal density and photosynthetic activity was found on the CO_2_ removal performance of the bubble column. On the contrary, a higher algae concentration in the circulating liquid imparted practical challenges of foaming, clogging, and increased oxygen concentrations in the upgraded biomethane. Although to achieve grid quality biomethane higher L/G ratios of 0.4 and above were necessary, the increased liquid flow rate resulted in a drop in the IC addition per unit volume into the alkaline algal liquid. In industrial-scale operations, this would not only lower the concentration of the cultivated microalgae but also impact the design of the PBR in terms of its hydraulic retention time and algal productivity. Thus, further optimization of the bubble column through the variation of gas and liquid flow rates, pH, alkalinity, temperature, and residence times would increase its integration and practicality in photosynthetic biogas upgrading.

## Footnotes

a Calculated using the fundamental equation Δ*G = −RT* ln *K*_eq_, where the equilibrium constant *K*_eq_ for the above reaction 19 is 2.6 × 10^–3^ at 25 °C.^54^
